# A Mechanistic Modeling Framework for Predicting Metabolic Interactions in Complex Mixtures

**DOI:** 10.1289/ehp.1103510

**Published:** 2011-08-11

**Authors:** Shu Cheng, Frederic Y. Bois

**Affiliations:** 1 Bioengineering Department, Royallieu Research Center, Université de Technology de Compiègne, Compiègne Cedex, France; 2 DRC/VIVA/METO Unit, Institut National de l’Environnement Industriel et des Risques (INERIS), Parc Technologique Alata, Verneuil en Halatte, France

**Keywords:** MCMC, metabolic interactions, PBPK model, reaction network, systems biology

## Abstract

Background: Computational modeling of the absorption, distribution, metabolism, and excretion of chemicals is now theoretically able to describe metabolic interactions in realistic mixtures of tens to hundreds of substances. That framework awaits validation.

Objectives: Our objectives were to *a*) evaluate the conditions of application of such a framework, *b*) confront the predictions of a physiologically integrated model of benzene, toluene, ethylbenzene, and *m*-xylene (BTEX) interactions with observed kinetics data on these substances in mixtures and, *c*) assess whether improving the mechanistic description has the potential to lead to better predictions of interactions.

Methods: We developed three joint models of BTEX toxicokinetics and metabolism and calibrated them using Markov chain Monte Carlo simulations and single-substance exposure data. We then checked their predictive capabilities for metabolic interactions by comparison with mixture kinetic data.

Results: The simplest joint model (BTEX interacting competitively for cytochrome P450 2E1 access) gives qualitatively correct and quantitatively acceptable predictions (with at most 50% deviations from the data). More complex models with two pathways or back-competition with metabolites have the potential to further improve predictions for BTEX mixtures.

Conclusions: A systems biology approach to large-scale prediction of metabolic interactions is advantageous on several counts and technically feasible. However, ways to obtain the required parameters need to be further explored.

Predicting the health effects of combined exposures from food, therapeutic treatments, and living or working environment is a long-standing challenge to toxicology. If additivity is assumed, toxic equivalency factors can be used (Walker et al. 2005). Otherwise the whole mixture, assuming that it is sufficiently well defined, can be tested as a single entity. Yet, that offers little mechanistic insight or predictive capacity for the vast majority of real-life mixed exposures. The challenge to mechanistic studies lies in accounting for the possible nonlinear interactions between substances at the pharmacokinetic, metabolic, or pharmacodynamic levels. They should also avoid the “curse of dimensionality,” which can affect experimental and modeling approaches. For example, assessing pairwise interactions between *n* substances requires a number of assays or analyses proportional to *n*(*n* – 1)/2, the number of pairs to examine. For higher-order interactions, between triples, quadruples, and so forth, the number of assays grows with the cube, the quartic, and so on, of *n*. To avoid the curse of dimensionality, one can stay within the limits of four to five substances in the mixtures considered. The published modeling literature does not venture much beyond those bounds (Haddad et al. 1999, 2000), even though realistic mixtures are much more complex.

To tackle mechanistically the question of realistic mixtures, we recently proposed a systems biology approach that, in its simplest form, addresses metabolic interactions (Bois 2009b, 2009c). Our approach uses the combined ingredients of generic (substance-independent) physiologically based pharmacokinetic (PBPK) modeling, systems biology markup language (SBML) modeling of metabolic networks, detailed (non-Michaelian) description of enzymatic reactions, and automatic code generation. Until now, we have used it only in a simulation complex, without confrontation with actual data. In this article, we present results of its application to mixtures of benzene, toluene, ethylbenzene, and *m*-xylene (BTEX), for which we have good data. We built and calibrated a predictive interaction model using single-chemical exposure data to obtain enzymatic microconstant values for benzene, toluene, ethylbenzene, and *m*-xylene individually. We then used the model to predict metabolic interactions in BTEX mixtures. We compared the resulting predictions with experimental mixture data and we explored the sensitivity of the predictions with respect to model structure.

## Materials and Methods

**Figure 1:**
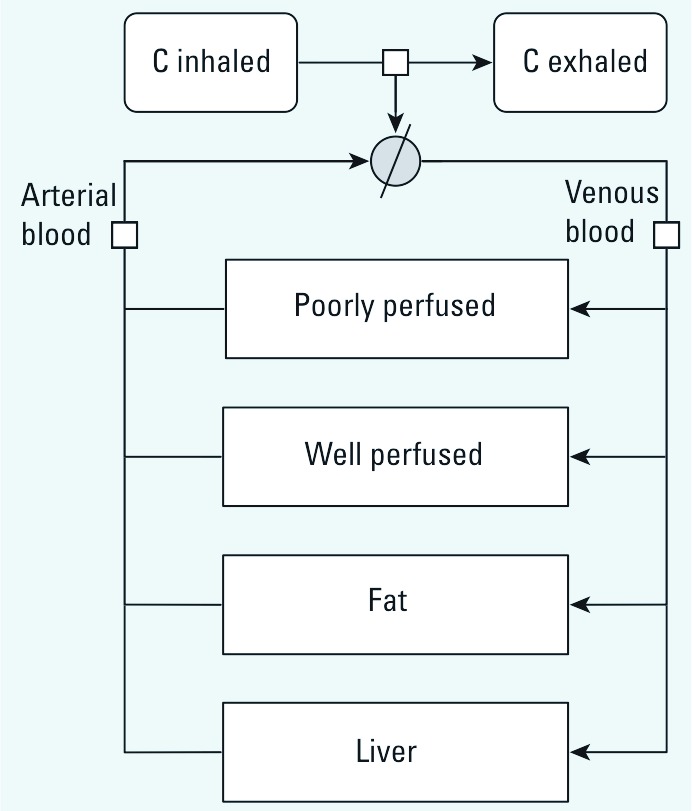
Generic rat PBPK template model used for each of the four BTEX constituents. C, concentration of BTEX constituent.

*PBPK model.* Figure 1 shows the structure of the generic PBPK rat model used for each of the four chemicals investigated. That model served as an automatic template for generating absorption, transport, and excretion terms in the global model equations; it does not describe metabolism. Substances were assumed to partition between the liver, fat, poorly perfused or well perfused tissues, exhaled air, arterial blood, and venous blood, as reported by Haddad et al. (1999). Only inhalation was modeled. The differential equations describing the time evolution of the quantity *Q_i_* of a substance in liver, fat, and poorly perfused or well perfused tissues were of the form


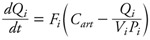
, [1]

where *F_i_* is blood flow through tissue *i* (*i* – 1, . . ., 4), *V_i_* is its volume, *P_i_* is the corresponding tissue/blood partition coefficient, and *C*_art_ is the arterial blood concentration. Concentration dynamics in venous blood (*C*_ven_), arterial blood (*C*_art_), and exhaled air (*C*_exh_) were described by algebraic equations:


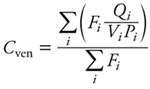
, [2]


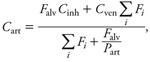
[3]


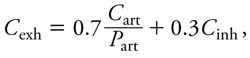
[4]

where *F*_alv_ is alveolar ventilation rate, *C*_inh_ is the inhaled substance concentration in air, and *P*_art_ is the blood/air partition coefficient. Baseline PBPK model parameters values are reported in Tables 1 and 2. Some parameters were treated as random variables and assigned statistical distributions as explained below, but their sampling distributions stayed centered on their baseline values.

**Table 1:** Chemical-independent model parameter baseline values.

Parameter	Value*a*
Total quantity of CYP2E1 (mmol)	2.09 × 10^–5b^
Alveolar ventilation rate (mL/min)	62.5*c*
Cardiac output (mL/min)	62.5*c*
Fraction of cardiac output to compartments	
Liver	0.25
Fat	0.09
Poorly perfused tissues	0.15
Richly perfused tissues	0.51*d*
Total body volume (mL)	250
Volume of compartments (mL)	
Liver	10
Fat	17.5*c*
Richly perfused tissues	12.5
Poorly perfused tissues	185*e*
**a**Data from U.S. Environmental Protection Agency (1988), except for CYP2E1 quantity. **b**Data from Carlile et al. (1997) and Seaton et al. (1995). **c**Subsequently sampled from statistical distributions (see “Materials and Methods”), using the given values as means. **d**Computed as 1.0 minus the sum of the other cardiac output fractions. **e**Computed as 0.9 times total body volume minus the sum of the other tissue volumes.	

**Table 2:** Chemical-dependent model parameters and baseline values.

Parameter	Benzene*a*	Toluene*b*	Ethylbenzene*b*	*m*-Xylene*b*
Partition coefficients								
Blood/air		15*c*		18*c*		42.7*c*		46*c*
Liver/blood		1.13		4.64		1.96		1.97
Fat/blood		33.3		56.7		36.4		40.4
Poorly perfused/blood		1.0		1.54		0.61		0.91
Richly perfused/blood		1.13		4.64		1.41		1.97
Reaction rate constants								
*k*_1_ (min^–1^ × nmol^–1^)		—*c*		—*c*		—*c*		—*c*
*k*_2_ (min^–1^)		—*c*		—*c*		—*c*		—*c*
*k*_3_ (min^–1^)		13.54*c*		10.38*c*		13.7*c*		10.32*c*
**a**Data from Travis et al. (1990), except for *k*_1_, *k*_2_, and *k*_3_ (from Dennison et al. 2003). **b**Data from Tardif et al. (1997), except for *k*_1_ and *k*_2_. **c**No set baseline value, parameter subsequently sampled from statistical distributions and estimated in this study (see Table 3 and “Materials and Methods”).								

**Figure 2:**
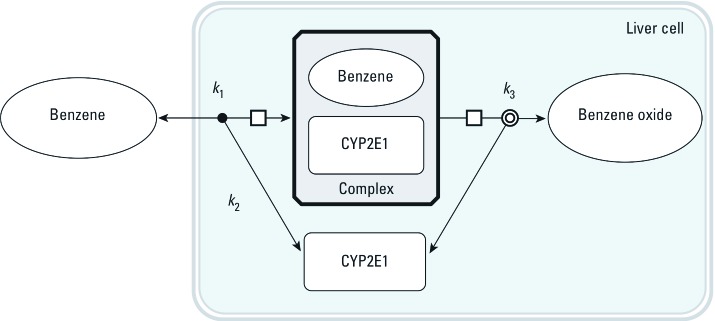
Graphical representation of the baseline SBML model used to describe benzene oxidation into benzene oxide by CYP2E1. Three rate constants, *k*_1_, *k*_2_, and *k*_3_, are used. Similar models were built for ethylbenzene, toluene, and *m*-xylene.

*SBML models of metabolic reactions.* For each chemical, the basic steps of its cytochrome P450 2E1 (CYP2E1)-catalyzed oxidation were coded in an SBML model (Hucka et al. 2003). Figure 2 shows the corresponding model for benzene. In rats, BTEX components are primarily oxidized by CYP2E1, giving rise to benzene oxide (Golding et al. 2010), phenylmethanol (or benzyl alcohol) (Nakajima 1997), 1-phenylethanol (Saghir et al. 2009), and (3-methylphenyl) methanol (or 3-methylbenzyl alcohol) (Nakajima 1997), respectively. Four basic SBML models (type I models, one for each chemical) were developed with the following four differential equations (using benzene, B, as an example):


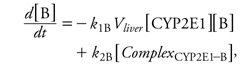
[5]


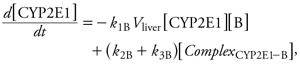
[6]


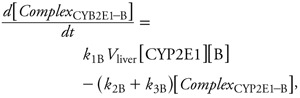
[7]


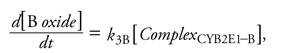
[8]

where *k*_1B_, *k*_2B_, and *k*_3B_ are the reactions’ microconstants for benzene. When computing, we actually used the quantity of CYP2E1 in the liver, which is the product of liver volume (*V*_liver_) and the CYP2E1 concentration in the above equations.

**Figure 3:**
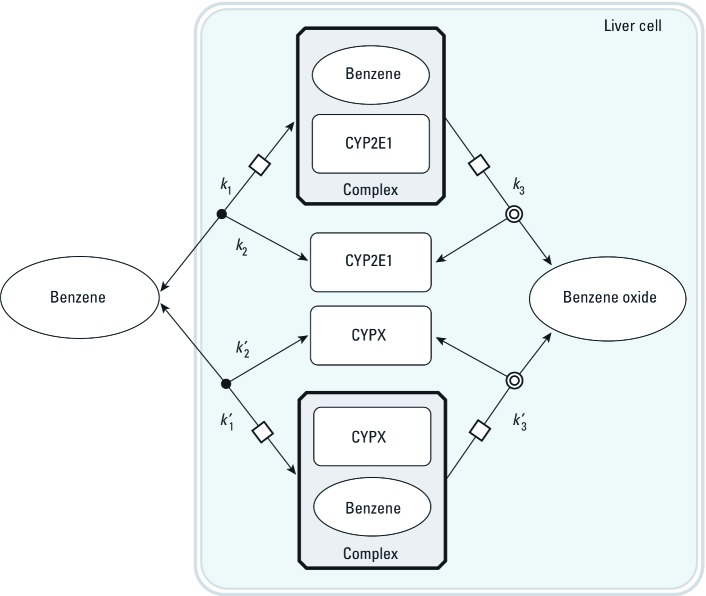
Graphical representation of the SBML model type II used to describe benzene oxidation into benzene oxide by two concurrent cytochromes. Six rate constants (*k*_1_, *k*_2_, *k*_3_, *k*_1_´, *k*_2_´, *k*_3_´) are needed. Similar models were built for ethylbenzene, toluene, and *m*-xylene.

**Figure 4:**
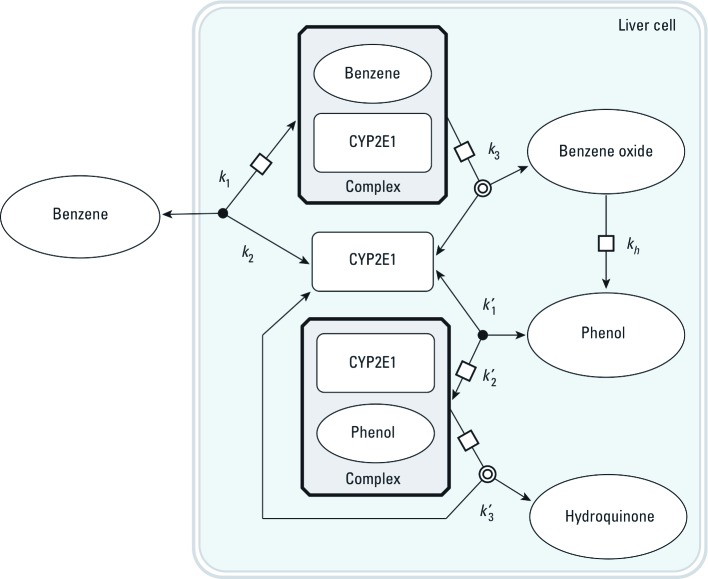
Graphical representation of the SBML model type III used to describe the competition between benzene and its metabolites for access to CYP2E1. Seven rate constants are needed. *k*_h_, first order rate constant for benzene oxide to phenol conversion. Similar models were built for ethylbenzene, toluene, and *m*-xylene.

Two sets of alternative SBML models (type II and type III models) were also coded to explore the effect of more complex metabolism. In type II models (Figure 3), benzene, toluene, ethylbenzene, or *m*-xylene can be metabolized by a second, unspecified enzyme, CYP*X*. The corresponding differential equations have additional terms, similar to those in Equations 5–8, for the new reactions. Type III models (Figure 4) considered the competition of the metabolites formed with their parents at the active site of CYP2E1. In all cases, the total quantities of CYP2E1 and CYP*X* enzymes were assumed to be constant over time.

*Automatic code generation for global model.* A C language file coding the complete set of differential equations describing the joint transport and metabolism of the four substances (“global model,” in the following) was automatically generated from the individual SBML metabolic pathway files and compiled by GNU MCSim (version 5.3.1; http://www.gnu.org/software/mcsim/). The transport terms, based on the PBPK template presented above, were generated for each chemical species placed outside the liver cell compartment in SBML. Species placed in the liver (e.g., CYP2E1) were not transported. Interactions implied by the competition for the same enzyme(s) are automatically taken into account; for example, in type I models, benzene, toluene, ethylbenzene, and *m*-xylene all bind competitively to CYP2E1 (Equations 5–8). Therefore, the differential equation for free CYP2E1 is given by


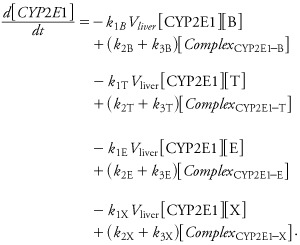
[9]

**Figure 5:**
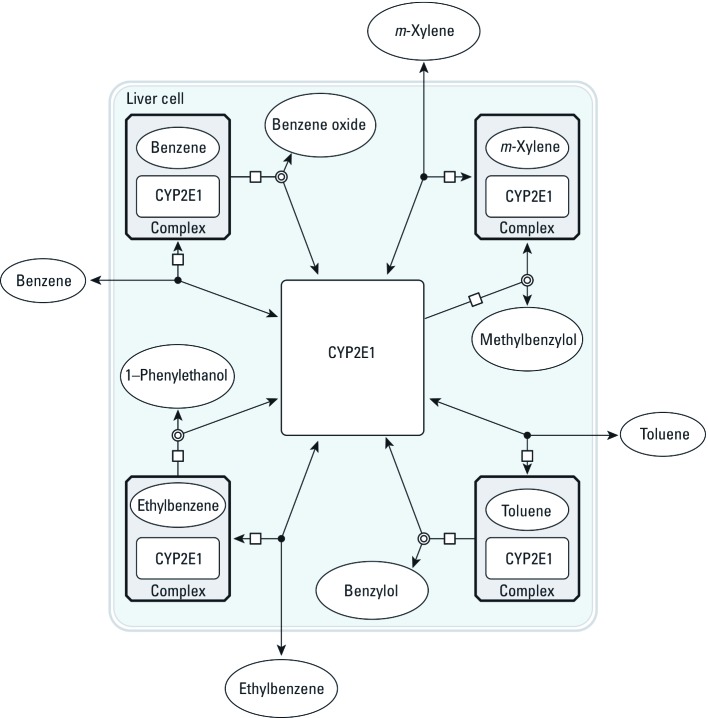
Graphical representation of the type I global metabolic interaction SBML model used for calibration and main predictions. BTEX components are circulated in the PBPK model shown in Figure 1. Their metabolites are not tracked by the model equations. Interactions occur through the depletion of the CYP2E1 pool.

The corresponding joint metabolic network of BTEX, without the PBPK part, is illustrated in Figure 5. With steady-state and rapid equilibrium assumptions, the full system of differential equations could be simplified to yield the usual interaction parameters *k_i_* as functions of the microconstants (Segel 1975). Such Michaelian treatment would be an approximation of our general solution, which does not require interaction parameters because metabolic interactions are automatically accounted for through the depletion of free CYP2E1.

*Type I model calibration and predictions.* The available literature only reports values for maximum velocity (*V*_max_) and the Michaelis constant (*K*_m_) (Haddad et al. 1999, 2001), whereas our formulation requires the microconstants *k*_1_, *k*_2_, and *k*_3_ for each component of BTEX. Indeed, *V*_max_, *K*_m_, *k*_1_, *k*_2_, and *k*_3_ are linked through classical relationships (Segel 1975):


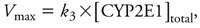
[10]


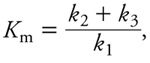
[11]

but knowledge of *V*_max_ and *K*_m_ leaves one of the microconstants unspecified. Therefore, we obtained values for *k*_1_, *k*_2_, and *k*_3_ by Bayesian calibration (Bernillon and Bois 2000) of the global model type I to kinetic data on venous blood concentrations of benzene, toluene, ethylbenzene, or *m*-xylene administered alone to rats (inhalation exposures to 50, 100, or 200 ppm in the air) (Haddad et al. 2000). We first performed a Monte Carlo sensitivity analysis of benzene data (as described by Haddad et al. 2000) to check which flows, volumes, and benzene-specific parameters needed to be calibrated. Most model parameters were sampled uniformly within ±10% of their baseline values (Tables 1, 2). Parameters *k*_1_, *k*_2_, and *k*_3_ were sampled uniformly in the intervals [10^6^, 10^7^], [5, 15], and [2, 25] respectively. We calculated correlations between sampled parameter values and predicted benzene venous concentration at each measurement time after cessation of exposure to 50, 100, 200, or 500 ppm of benzene in the air. Parameters with at least one correlation coefficient absolute value exceeding 0.3 were regarded as sensitive. The results [Supplemental Material, Tables S1, S2 (http://dx.doi.org/10.1289/ehp.1103510)] indicated that, besides *k*_3_, the volume of fat, alveolar ventilation rate, cardiac output, and blood/air partition coefficient were sensitive. These parameters were therefore treated as random variables and sampled. Given the similarity of the kinetics of benzene, toluene, ethylbenzene, and *m*-xylene, we fitted the same parameters for the other three BTEX components as well.

Bayesian calibration was performed using Markov chain Monte Carlo (MCMC) simulations. The volume of fat, alveolar ventilation rate, cardiac output, and blood/air partition coefficient were sampled according to informative prior distributions (Table 3). Parameters *k*_1_ and *k*_2_ were sampled from wide uniform distributions, with informative bounds given by Segel (1975, p. 32). For *k*_3_ we used a narrower range because we had reasonable prior values for benzene, toluene, ethylbenzene, and *m*-xylene *V*_max_ (Bois and Paxman 1992; Dennison et al. 2003; Tardif et al. 1997) and for the total quantity of CYP2E1 in liver, hence for its total liver concentration [CYP2E1]_total_ (Carlile et al. 1997; Seaton et al. 1995).

**Table 3:** Prior distributions and posterior distribution summaries of the model parameters calibrated by MCMC sampling.

Parameter	Prior distribution	Posterior distribution*a*
Alveolar ventilation rate (mL/min)		LN(62.5, 1.1)		78; 78 ± 5.6 (67, 89)
Cardiac output (mL/min)		LN(62.5, 1.1)		90; 88 ± 6.8 (75, 100)
Volume of fat (mL)		LN(17.5, 1.1)		12; 11.7 ± 0.8 (10, 13)
Blood/air partition coefficients				
Benzene		LN(15, 1.1)		15.4; 16 ± 1.2 (14, 19)
Toluene		LN(18, 1.1)		17.2; 18 ± 1.7 (15, 22)
Ethylbenzene		LN(42.7, 1.1)		40.3; 43 ± 4.0 (36, 52)
*m*-Xylene		LN(46, 1.1)		48.5; 47 ± 4.5 (38, 56)
*k*_1_ rate constant (min^–1^ × nmol^–1^)				
Benzene		U(10^–3^, 10^2^)		39; 66 ± 22 (20, 98)
Toluene		U(10^–3^, 10^2^)		26; 66 ± 22 (20, 98)
Ethylbenzene		U(10^–3^, 10^2^)		94; 65 ± 23 (19, 98)
*m*-Xylene		U(10^–3^, 10^2^)		61; 65 ± 22 (21, 98)
*k*_2_ rate constant (min^–1^)				
Benzene		U(10, 10^7^)		1,040; 1,900 ± 900 (480, 3,900)
Toluene		U(10, 10^7^)		2,900; 8,400 ± 4,500 (1,700, 19,000)
Ethylbenzene		U(10, 10^7^)		22,000; 17,000 ± 6,600 (4,500, 29,000)
*m*-Xylene		U(10, 10^7^)		6,800; 9,000 ± 4,000 (2,400, 1,8000)
*k*_3_ rate constant (min^–1^)				
Benzene		U(2, 50)		7.8; 8.3 ± 1.0 (6.4, 10)
Toluene		U(2, 50)		12; 13 ± 1.6 (10, 16)
Ethylbenzene		U(2, 50)		17; 18 ± 1.8 (15, 22)
*m*-Xylene		U(2, 50)		18; 19.5 ± 2.5 (15, 25)
Abbreviations: LN, lognormal; U, uniform. **a**Values shown are mode; mean ± SD (2.5th percentile, 97.5th percentile).				

The data were assumed to be log-normally distributed with a geometric mean predicted by the model and a geometric standard deviation (SD) computed from the arithmetic SD given for each data point by Haddad et al. (2000). The geometric SDs had an average of 1.28. Hence variability appeared small in this data set, and we used the average concentrations (for five rats) for each time point as data.

To obtain samples from the joint posterior distribution of the model parameters, five Markov chains were run independently for each chemical. Their convergence was assessed using Gelman and Rubin’s (1992) *R* diagnostic.

As a cross-validation, the last step was to perform posterior predictive simulations of the kinetics of benzene, toluene, ethylbenzene, and *m*-xylene administered together in various mixture combinations. We simulated the mixture dosing conditions of Haddad et al. (1999) and Tardif et al. (1996) with the global model and parameter values from the joint posterior sample obtained by MCMC simulations. To simulate experimental error and interindividual variability, we added a log-normal noise with a geometric mean of 1 and geometric SD of 1.28, consistent with the above error model.

*Type II and III model predictions.* Those models are more complex and each require 12 additional reaction microconstants. We had no hope of identifying them with the data available and therefore set them to plausible values. For the parameters common to all three models (e.g., organ volumes), we used the posterior values of global model I (Tables 1–3). For global model II, *k*_2_´ and *k*_3_´ for CYP*X* were set at the same values (or distribution) as for CYP2E1, but CYP*X k*_1_´ was set to 1/10 of CYP2E1 *k*_1_. CYP*X* therefore behaves as a lower affinity, lower capacity pathway than CYP2E1. The total quantity of CYP*X* in rat liver was set at 1.16 × 10^–6^ mmol, similar to that of CYP2B1 (Ngui and Bandiera 1999). For the type III global model, the parameters of the metabolites were set equal to those of the parents.

*Software used.* SBML metabolism models were individually coded using CellDesigner® (version 4.1; Funahashi et al. 2003). GNU MCSim (version 5.3.1; Bois 2009a; Bois and Maszle 1997) was used to build the PBPK model template and the global model and for the computations. R software (version 2.11.0; R Development Core Team 2010) was used for graphics. The SBML files, the PBPK template, and the global model files are provided as Supplemental Material (http://dx.doi.org/10.1289/ehp.1103510).

## Results

**Figure 6:**
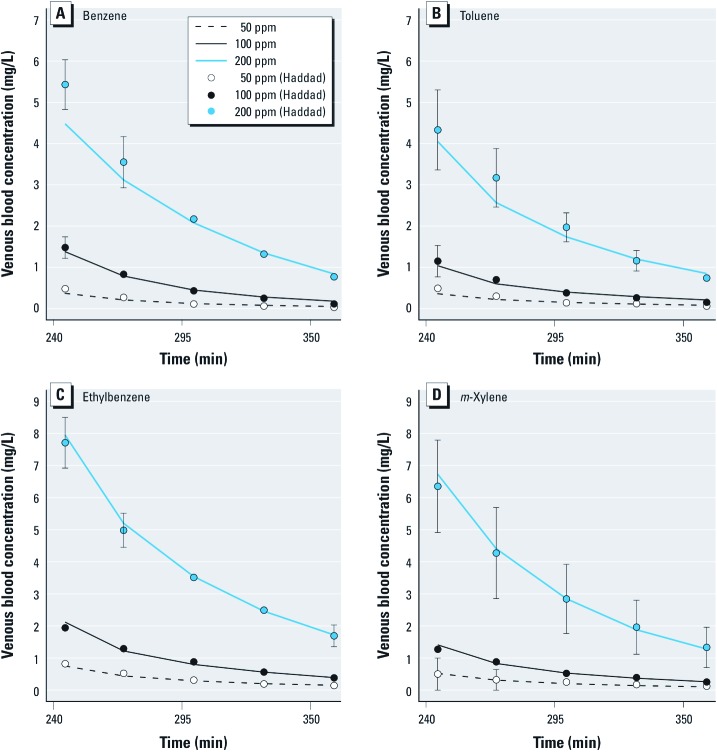
Maximum posterior fit of the type I global model to venous blood concentration data [means ± SDs (data from Haddad et al. 2000)] on benzene (*A*), toluene (*B*), ethylbenzene (*C*), and *m*-xylene (*D*) in rats after a single chemical exposure to 50 ppm, 100 ppm, or 200 ppm.

*Model calibration using individual benzene, toluene, ethylbenzene, or* m*-xylene exposures.* Global model type I (see Figures 1, 2, 5) was calibrated on the basis of rat exposures to single substances (Haddad et al. 2000). Venous blood concentration data were measured at five time points during the 2 hr after a 4-hr inhalation exposure to 50, 100, or 200 ppm of each substance. Convergence of the five MCMC chains was obtained after 100,000 iterations (*R* diagnostic < 1.021 for any parameter). The following results were obtained using 10,000 joint posterior parameter samples. Table 3 summarizes the posterior distributions. Most parameters were well identified, with larger uncertainties about the *k*_1_ and *k*_2_ rate constants (about 20–50% coefficient of variation). We used Equation 11 to compute *K*_m_ values corresponding to the sampled *k*_1_, *k*_2_, and *k*_3_ values and compared them with previously published values [see Supplemental Material, Table S3 (http://dx.doi.org/10.1289/ehp.1103510)]. Figure 6 shows the maximum posterior fit of the type I global model to the single-exposure data.

**Figure 7:**
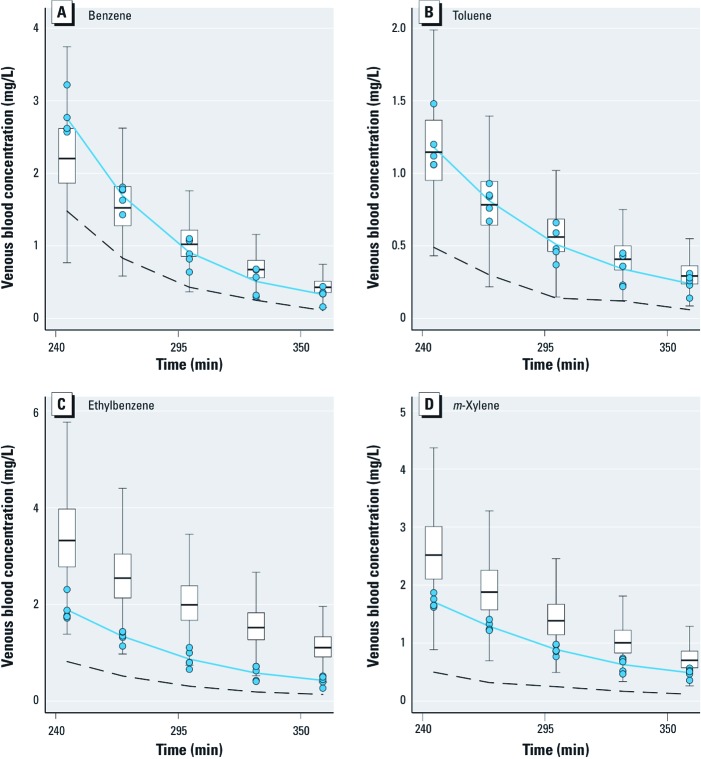
Blood kinetics of benzene (*A*), toluene (*B*), ethylbenzene (*C*), and *m*-xylene (*D*) after exposure to a quaternary mixture of 100 ppm of benzene and 50 ppm of each of the other three BTEX components. The box plots display the interquartile range (IQR) of the type I global model predictions (without any fitting to the mixture data). The boxes correspond to the 25th and 75th percentiles, the horizontal lines within the boxes indicate median concentrations and the upper end of whisker is the value below 75th percentile plus 1.5 × IQR, while the lower end of whisker is the value above 25th percentile minus 1.5 × IQR. The blue circles represent experimental data for five rats, and the solid blue lines give their means (data from Haddad et al. 1999). The dashed lines indicate mean experimental venous concentrations of each chemical when administered alone at the same level in air (i.e., without interactions) (data from Haddad et al. 2000).

*Model predictions and cross-validation for BTEX mixtures.* Figure 7 shows box plots of the type I global model predictions of BTEX venous blood concentrations in rats after a quaternary mixture exposure (4 hr to 100 ppm benzene and 50 ppm for the other three compounds). The corresponding data (Haddad et al. 1999) are overlaid for comparison. These data were not used for model calibration and provide an independent model check. The concentrations of different chemicals are significantly increased (by about a factor 2) compared with those obtained after similar single exposures to the substances. Model predictions consistently overlap the data, and the interactions are qualitatively forecasted correctly. The predictions are very good for benzene and toluene but overshoot the data for ethylbenzene and *m*-xylene. On average, the data means and prediction medians differ by about 25%. We also performed predictions for binary 100 ppm toluene and 200 ppm *m*-xylene coexposures or ternary toluene, ethylbenzene, and *m*-xylene coexposures (100 ppm each) (Tardif et al. 1996). The results were similar to those of Figure 7 [Supplemental Material, Figures S1, S2 (http://dx.doi.org/10.1289/ehp.1103510)].

*Exploration of modeling alternatives.* In order to check whether the model predictions would be altered significantly by the addition of a minor metabolic pathway or by the possibility of competition with the metabolites formed, we studied type II and type III global models (see Figures 3, 4). Using model type II had little impact on the predictions. Model type III was able to partly improve them, in some cases removing or even reversing the biases seen in Figure 7. {Results of this analysis [see Supplemental Material, Figures S3–S8 (http://dx.doi.org/10.1289/ehp.1103510)] and model and input files that can be used to perform new simulations with GNU MCSim are available at http://www.gnu.org/software/mcsim/supplement_EHP_2011.tar.gz.} Neither model gave a consistent improvement in predictions, but with adequate parameter values they could potentially lead to a refined prediction of interactions.

## Discussion

We used a modeling framework theoretically able to predict complex metabolic interactions among an unlimited number of drugs, environmental and workplace contaminants, and food-borne natural chemicals (Bois 2009b, 2009c). The first component of this approach is to integrate it in a PBPK framework. It is well known that metabolic interactions are nonlinear phenomena that depend on internal concentrations at the site of metabolism (Haddad et al. 2001; Yang 2010; Yang et al. 1995). PBPK models are becoming generic, at least in their transport components, and offer a consistent solution to that question even when a large number of substances are investigated jointly (Bois et al. 2010; Bouvier d’Yvoire et al. 2007). Still, metabolic pathway modeling is not yet generic, even though it is an important component of PBPK models. The development of a library of quantitative SBML models for substances of general interest, along the lines presented here, could provide a versatile solution to that problem. SBML (Hucka et al. 2003) is one option among many, but it is well supported and is increasingly used by the systems biology community. SBML is also a high-level language developed explicitly to provide a common intermediate format for representing and exchanging models between simulation or analysis tools. That feature has greatly facilitated the development of our software for the automatic generation of coupled model equations from a generic PBPK template and a library of SBML models (Bois 2009a). With the current computing capabilities of personal computers, a mixture model for about 100 substances can simulate a full day in a few seconds of computer time.

The use of reaction microconstants is an important feature of our approach. This may be seen as a step back to pre-Michaelian enzyme kinetics, but there are three arguments in its favor. First, Michaelis-Menten kinetics are an approximation (Bardsley et al. 1980; Hill et al. 1977), and current computing and experimental capabilities do not need to rely on an approximation. Second, microconstant formulations permit a prediction of interactions from first principles (Luecke and Wosilait 1979) without resorting to empirical interaction constants (usually denoted as *k_i_*) for each pair of chemicals considered. That simplifies modeling considerably: to model any mixture, it is enough to draw the individual metabolic schemes of its constituents and write their joint equation system. The same metabolic pathways and parameters values can be reused for different mixtures. Finally, our approach is in the long run more parsimonious in terms of parameters. The Michaelis-Menten formulation requires two parameters per chemical and per reaction (*V*_max_ and *K*_m_) and usually two *k_i_* for each pair of interacting substances. The number of *k_i_* parameters therefore grows with the square of the number *n* of substances in the mixture. Our approach needs only three microconstants (*k*_1_, *k*_2_, and *k*_3_) per chemical and per reaction, whatever the complexity of the mixture, and the number of parameters grows only linearly with it. With four substances, 20 parameters are classically “needed,” whereas our approach requires only 12. The corresponding figures for a mixture of 50 substances are 2,550 versus 150. A little used implication of the Michaelian competitive inhibition model is that the parameters *k_i_* are equal to the *K*_m_ of each chemical (Luecke and Wosilait 1979), but that is still an approximation, without the flexibility to go beyond simple competition or the simplicity of our model building approach.

Yet, the use of fundamental constants has drawbacks. Although values for *V*_max_, *K*_m_, and *k_i_* are routinely available, values for *k*_1_, *k*_2_, and *k*_3_ are not; we had to obtain them using model calibration. Microconstant values could be obtained from specific experimental protocols or quantum chemistry modeling (work in progress in our laboratory). The Michaelis-Menten approximation also gives reasonable results in many cases when studying one or two substances. It is interesting to note that the question of interactions has been reduced in the drug industry to that of “drug–drug” interactions, even if that is a severe reduction of the whole question. Finally, experimentally determined *k_i_* do not need to describe a precise mechanism and can approximate a variety of them. In contrast, our approach requires a precise definition of the hypothesized interaction mechanisms.

To demonstrate our approach, we confronted its predictions with well-studied data. The BTEX case, even if on a limited number of chemicals, is important and has been well studied (Dennison et al. 2003; Haddad et al. 1999; Tardif et al. 1996, 1997). It was difficult, however, to obtain precise values for *k*_1_, *k*_2_, and *k*_3_ from whole-body kinetic data (see Figure 6, Table 3). This comes as little surprise because Michaelis and Menten developed their simplification precisely to address that difficulty. We were able to set an informative prior for *k*_3_ on the basis of published *V*_max_ values for benzene, toluene, ethylbenzene, and *m*-xylene. For *k*_1_ and *k*_2_, we resorted to wide ranges, with upper bounds imposed by physical constraints on diffusions (Segel 1975). Their ratio, however, is reasonably well identified. The model fit after calibration is excellent, and little would be gained by a multilevel analysis (Bois et al. 1996). In addition, the *K*_m_ values implied by the posterior distributions of the microconstants are reasonably close to previous estimates [see Supplemental Material, Table S3 (http://dx.doi.org/10.1289/ehp.1103510)].

Overall, our results show that a microconstant-based model (model I) gives a correct qualitative picture of the interactions observed in BTEX mixtures. Quantitatively, about 50% of the interaction effects are predicted. We did not have sufficient information to precisely calibrate improved models of BTEX metabolism. We know, however, that such models surely exist. The initial steps of benzene metabolism involve CYP-dependent oxidation. CYP2E1 and to some extent CYP2B1 are likely to be the main enzymes for benzene metabolism at high levels of exposure (Gut et al. 1996; Nakajima 1997). A second high-affinity, low-capacity CYP2F1 or CYP2A13 pathway has been proposed (Rappaport et al. 2009, 2010). We performed a sensitivity analysis of the impact of model structure with models II and III, which include alternative pathways and competitions between parents and metabolites. Accounting for minor pathways and secondary metabolism could clearly improve predictions of BTEX interactions, but we did not get dramatic changes indicative of a strong sensitivity to secondary phenomena. Our description of the liver is also simplistic and could be improved with a finer description of liver zonation-dependent metabolism (Andersen et al. 1997; Sheikh-Bahaei et al. 2009; Wambaugh and Shah 2010). It would not be difficult to extend our models with more enzymes and complex reactions mechanisms (including enzymatic induction) (Bois 2009c; Luke et al. 2010) or specific early toxicity pathways.

## Conclusions

A first-principles, systems biology, or mechanism-based approach for large-scale prediction of metabolic interactions is technically feasible and worth exploring. Generic PBPK models are an essential ingredient of it. The predictions made here for the BTEX case were basically correct, if not perfect. Better experimental or modeling ways to obtain kinetic microconstants should be explored. This agenda applies to systems biology as a whole, and tools are becoming available to calibrate cell-level models (Bois 2009a; Vyshemirsky and Girolami 2008). The major conclusion of this work is that detailed quantitative understanding of the metabolic and toxicity pathways of individual chemicals should be sufficient to predict interactions in complex mixtures. Using the framework we present here, predictive models for arbitrary mixtures can be automatically generated on the basis of a library of models for individual substances. That approach is entirely congruent with trend to develop and share modular libraries of systems biology models (Le Novere et al. 2006).

## Supplemental Material

(315 KB) PDF

## References

[r1] Andersen ME, Birnbaum LS, Barton HA, Eklund CR (1997). Regional hepatic CYP1A1 and CYP1A2 induction with 2,3,7,8-tetrachlorodibenzo-p-dioxin evaluated with a multicompartment geometric model of hepatic zonation.. Toxicol Appl Pharmacol.

[r2] Bardsley WG, Leff P, Kavanagh J, Waight RD (1980). Deviations from Michaelis-Menten kinetics.. Biochem J.

[r3] Bernillon P, Bois FY (2000). Statistical issues in toxicokinetic modeling: a Bayesian perspective.. Environ Health Perspect.

[r4] Bois FY (2009a). GNU MCSim: Bayesian statistical inference for SBML-coded systems biology models.. Bioinformatics.

[r5] Bois FY (2009b). Modélisation physiologique des interactions métaboliques. Env Risque Santé.

[r6] Bois FY (2009c). Physiologically-based modelling and prediction of drug interactions.. Basic Clin Pharmacol Toxicol.

[r7] Bois FY, Jackson E, Pekari K, Smith M (1996). Population toxicokinetics of benzene.. Environ Health Perspect.

[r8] Bois F, Jamei M, Clewell HJ (2010). PBPK modelling of inter-individual variability in the pharmacokinetics of environmental chemicals.. Toxicology.

[r9] Bois FY, Maszle D (1997). MCSim: a simulation program.. J Stat Softw.

[r10] Bois FY, Paxman D (1992). An analysis of exposure rate effects for benzene using a physiologically based pharmacokinetic model.. Regul Toxicol Pharmacol.

[r11] Bouvier d’Yvoire M, Prieto P, Blaauboer BJ, Bois FY, Boobis A, Brochot C (2007). Physiologically-based kinetic modelling (PBK modelling): meeting the 3Rs agenda—the report and recommendations of ECVAM Workshop 63a.. Altern Lab Anim.

[r12] Carlile DJ, Zomorodi K, Houston JB (1997). Scaling factors to relate drug metabolic clearance in hepatic microsomes, isolated hepatocytes, and the intact liver: studies with induced livers involving diazepam.. Drug Metab Dispos.

[r13] Dennison JE, Andersen ME, Yang RS (2003). Characterization of the pharmacokinetics of gasoline using PBPK modeling with a complex mixtures chemical lumping approach.. Inhal Toxicol.

[r14] Funahashi A, Tanimura N, Morohashi M, Kitano H. (2003). CellDesigner: a process diagram editor for gene-regulatory and biochemical networks.. Biosilico.

[r15] Gelman A, Rubin DB (1992). Inference from iterative simulation using multiple sequences (with discussion).. Stat Sci.

[r16] Golding BT, Barnes ML, Bleasdale C, Henderson AP, Jiang D, Li X (2010). Modeling the formation and reactions of benzene metabolites.. Chem Biol Interact.

[r17] Gut I, Nedelcheva V, Souèek P, Stopka P, Vodièka P, Gelboin HV (1996). The role of CYP2E1 and 2B1 in metabolic activation of benzene derivatives.. Arch Toxicol.

[r18] Haddad S, Béliveau M, Tardif R, Krishnan K. (2001). A PBPK modeling-based approach to account for interactions in the health risk assessment of chemical mixtures.. Toxicol Sci.

[r19] Haddad S, Charest-Tardif G, Tardif R, Krishnan K. (2000). Validation of a physiological modeling framework for simulating the toxicokinetics of chemicals in mixtures.. Toxicol Appl Pharmacol.

[r20] Haddad S, Tardif R, Charest-Tardif G, Krishnan K. (1999). Physiological modeling of the toxicokinetic interactions in a quaternary mixture of aromatic hydrocarbons.. Toxicol Appl Pharmacol.

[r21] Hill CM, Waight RD, Bardsley WG (1977). Does any enzyme follow the Michaelis-Menten equation?. Mol Cell Biochem.

[r22] Hucka M, Finney A, Sauro HM, Bolouri H, Doyle JC, Kitano H (2003). The systems biology markup language (SBML): a medium for representation and exchange of biochemical network models.. Bioinformatics.

[r23] Le Novere N, Bornstein B, Broicher A, Courtot M, Donizelli M, Dharuri H (2006). Biomodels Database: a free, centralized database of curated, published, quantitative kinetic models of biochemical and cellular systems.. Nucleic Acids Res.

[r24] Luecke RH, Wosilait WD (1979). Drug elimination interactions: analysis using a mathematical model.. J Pharmacokinet Biopharm.

[r25] Luke NS, DeVito MJ, Shah I, El-Masri HA (2010). Development of a quantitative model of pregnane X receptor (PXR) mediated xenobiotic metabolizing enzyme induction.. Bull Math Biol.

[r26] Nakajima T. (1997). Cytochrome P450 isoforms and the metabolism of volatile hydrocarbons of low relative molecular mass.. J Occup Health.

[r27] Ngui JS, Bandiera SM (1999). Induction of hepatic CYP2B is a more sensitive indicator of exposure to Aroclor 1260 than CYP1A in male rats.. Toxicol Appl Pharmacol.

[r28] R Development Core Team (2010). R: A Language and Environment for Statistical Computing. Vienna:R Foundation for Statistical Computing.. http://www.R-project.org.

[r29] Rappaport SM, Sungkyoon K, Qing L, Guilan L, Vermeulen R, Waidyanatha S (2010). Human benzene metabolism following occupational and environmental exposures.. Chem Bioll Interact.

[r30] Rappaport SM, Sungkyoon K, Qing L, Vermeulen R, Waidyanatha S, Zhang L (2009). Evidence that humans metabolize benzene via two pathways.. Environ Health Perspect.

[r31] Saghir SA, Rick DL, McClymont EL, Zhang F, Bartels MJ, Bus JS (2009). Mechanism of ethylbenzene-induced mouse-specific lung tumor: metabolism of ethylbenzene by rat, mouse, and human liver and lung microsomes.. Toxicol Sci.

[r32] Seaton MJ, Follansbee MH, Bond JA (1995). Oxidation of 1,2-epoxy-3-butene to 1,2:3,4-diepoxybutane by cDNA-expressed human cytochromes P450 2E1 and 3A4 and human, mouse and rat liver microsomes.. Carcinogenesis.

[r33] Segel IH (1975).

[r34] Sheikh-Bahaei S, Kim SHJ, Sheikhbahaei S, Hunt CA (2009). Understanding the role of liver zonation in toxin elimination.. Int J Intell Control Syst.

[r35] Tardif R, Charest-Tardif G, Brodeur J. (1996). Comparison of the influence of binary mixtures versus a ternary mixture of inhaled aromatic hydrocarbons on their blood kinetics in the rat.. Arch Toxicol.

[r36] Tardif R, Charest-Tardif G, Brodeur J, Krishnan K. (1997). Physiologically based pharmacokinetic modeling of a ternary mixture of alkyl benzenes in rats and humans.. Toxicol Appl Pharmacol.

[r37] Travis CC, Quillen JL, Arms A (1990). Pharmacokinetics of benzene.. Toxicol Appl Pharmacol.

[r38] U.S. Environmental Protection Agency (1988). Reference Physiological Parameters in Pharmacokinetic Modeling. EPA/600/6-88/004.

[r39] Vyshemirsky V, Girolami M. (2008). BioBayes: a software package for Bayesian inference in systems biology.. Bioinformatics.

[r40] Walker NJ, Crockett PW, Nyska A, Brix AE, Jokinen MP, Sells DM (2005). Dose-additive carcinogenicity of a defined mixture of “dioxin-like compounds.”. Environ Health Perspect.

[r41] Wambaugh J Shah I. 2010 Simulating microdosimetry in a virtual hepatic lobule. PLoS Comput Biol 6; doi:10.1371/journal.pcbi.1000756 [Online 22 April 2010] PMC2858695 20421935

[r42] Yang RSH (2010). Toxicological interactions of chemical mixtures.

[r43] Yang RSH, El-Masri HA, Thomas RS, Constan AA (1995). The use of physiologically-based pharmacokinetic/pharmacodynamic dosimetry models for chemical mixtures.. Toxicol Lett.

