# Supplementary material for: A Mechanistic Modeling Framework for Predicting Metabolic Interactions in Complex Mixtures
Source: Environ Health Perspect. 2011 Aug 11;119(12):1712–8. doi: 10.1289/ehp.1103510 (PMC3261979; doi:10.1289/ehp.1103510)
Supplement: (315 KB) PDF [file ehp.1103510.s001.pdf]

Supplemental Material

A Mechanistic Modeling Framework for Predicting Metabolic Interactions in Complex Mixtures

Shu Cheng, Frederic Y. Bois

Table of Contents

Table S1: Results of the preliminary sensitivity analysis following cessation of exposure to 50 ppm or 100 ppm of benzene in the air 3

Table S2: Results of the preliminary sensitivity analysis following cessation of exposure to 200 ppm or 500 ppm of benzene in the air 4

Table S3. Comparison of km values (in mg/L) derived by MCMC calibration to those previously published........ 5

Figure S1: Blood kinetics of toluene (A) and m-xylene (B) after exposure to a binary mixture of 100 ppm of toluene and 200 ppm of m-xylene 6

Figure S2: Blood kinetics of toluene (A), ethylbenzene (B) and m-xylene (C) after exposure to a ternary mixture of 100 ppm of each 7

References........ 8

Additional Figures (S3-S8) and all model and input files to redo the simulations with *GNU MCSim* are available at http://www.gnu.org/software/mcsim/supplement_EHP_2011.tar.gz

Table S1: Results of the preliminary sensitivity analysis: Correlations between sampled parameter values and model predictions of benzene venous blood concentration at each measurement time (5 min, 30 min, 60 min, 90 min and 120 min) following cessation of exposure to 50 ppm or 100 ppm of benzene in the air. Coefficients superior or equal to 0.3 are in bold.

Parameters	Correlations at 50 ppm exposure					Correlations at 100 ppm exposure			
	5min	30min	60min	90min	120min	5min	30min	60min	90min
k3	**-0.44**	**-0.42**	**-0.40**	**-0.38**	**-0.35**	**-0.69**	**-0.66**	**-0.62**	**-0.60**
k1	-0.07	-0.08	-0.09	-0.09	-0.09	-0.04	-0.04	-0.04	-0.05
k2	0.01	0.02	0.03	0.03	0.03	0.00	0.00	0.00	0.01
Body_V	0.02	0.11	0.12	0.11	0.09	0.00	0.06	0.07	0.06
V_fat	-0.09	-0.11	-0.03	0.08	0.19	-0.10	-0.10	-0.06	0.00
V_liv	-0.01	-0.01	-0.02	-0.02	-0.02	0.00	-0.01	-0.01	0.00
V_wp	0.19	-0.02	-0.08	-0.07	-0.05	0.13	-0.01	-0.04	-0.04
Pct_Flow_fat	0.02	0.05	0.07	0.06	0.05	0.01	0.03	0.04	0.04
Pct_Flow_liv	-0.08	-0.10	-0.13	-0.15	-0.15	-0.03	-0.03	-0.04	-0.05
Pct_Flow_pp	0.05	0.01	-0.02	-0.02	-0.02	0.04	0.02	0.00	0.00
Flow_alv	**0.42**	**0.36**	**0.37**	**0.37**	**0.36**	**0.34**	0.25	0.23	0.22
Flow_tot	-0.25	-0.28	**-0.35**	**-0.43**	**-0.52**	-0.08	-0.05	-0.06	-0.09
Benzene_PC_art	0.09	0.11	0.12	0.13	0.13	0.10	0.11	0.12	0.12
Benzene_PC_fat	-0.02	-0.02	0.00	0.02	0.04	-0.02	-0.02	-0.02	0.00
Benzene_PC_liv	-0.01	-0.01	-0.01	-0.02	-0.02	0.00	0.00	0.00	-0.01
Benzene_PC_wp	0.01	0.01	0.00	0.00	0.00	0.00	-0.01	-0.01	-0.01
Benzene_PC_pp	0.00	0.03	0.04	0.04	0.03	-0.01	0.01	0.02	0.02

Table S2: Results of the preliminary sensitivity analysis: Correlations between sampled parameter values and model predictions of benzene venous blood concentration at each measurement time (5 min, 30 min, 60 min, 90 min and 120 min) following cessation of exposure to 200 ppm or 500 ppm of benzene in the air. Coefficients superior or equal to 0.3 are in bold.

Parameters	Correlations at 200 ppm exposure					Correlations at 500 ppm exposure			
	5min	30min	60min	90min	120min	5min	30min	60min	90min
k3	**-0.82**	**-0.83**	**-0.80**	**-0.77**	**-0.74**	**-0.63**	**-0.75**	**-0.81**	**-0.85**
k1	-0.02	-0.02	-0.03	-0.03	-0.03	0.00	0.00	-0.01	-0.01
k2	0.00	-0.01	-0.01	-0.01	-0.01	0.00	-0.01	-0.01	-0.01
Body_V	-0.02	0.04	0.06	0.05	0.04	-0.06	0.04	0.07	0.07
V_fat	-0.15	-0.16	-0.12	-0.06	0.00	**-0.30**	**-0.32**	-0.25	-0.16
V_liv	0.00	-0.01	-0.01	-0.01	0.00	0.00	0.00	-0.01	-0.01
V_wp	0.13	0.01	-0.03	-0.03	-0.03	0.21	0.06	-0.01	-0.02
Pct_Flow_fat	0.01	0.03	0.04	0.04	0.03	0.02	0.06	0.07	0.07
Pct_Flow_liv	-0.01	-0.01	-0.01	-0.02	-0.02	0.01	0.01	0.00	0.00
Pct_Flow_pp	0.04	0.03	0.01	0.00	0.00	0.05	0.04	0.01	0.01
Flow_alv	**0.39**	0.26	0.21	0.17	0.15	**0.53**	**0.33**	0.22	0.15
Flow_tot	0.02	0.06	0.06	0.05	0.02	0.09	0.20	0.23	0.21
Benzene_PC_art	0.15	0.16	0.16	0.16	0.16	**0.33**	**0.35**	**0.33**	**0.31**
Benzene_PC_fat	-0.03	-0.03	-0.02	-0.01	0.00	-0.06	-0.06	-0.05	-0.03
Benzene_PC_liv	0.01	0.01	0.00	0.00	0.00	0.02	0.01	0.01	0.01
Benzene_PC_wp	-0.01	-0.01	-0.01	-0.02	-0.02	0.00	0.00	-0.01	-0.01
Benzene_PC_pp	-0.01	0.00	0.01	0.01	0.01	-0.02	0.02	0.03	0.02

Table S3. Comparison of k_m values (in mg/L) derived by MCMC calibration to those previously published.

**Chemicsal**	**Posterior MCMC estimate** [a]	**Previous estimates**
Benzene	0.21, 0.23 ± 0.07 [0.11, 0.38]	0.1; 0.1; 0.2 [b]
Toluene	1.0, 1.2 ± 0.49 [0.40, 2.3]	0.55; 0.13; 0.02 [c]
Ethylbenzene	2.5, 2.75 ± 0.44 [2.0, 3.7]	1.39; 1.04; 0.1 [c]
m-Xylene	1.2, 1.5 ± 0.4 [0.81, 2.4]	0.22; 0.45 [d]

[a] Mode, mean ± SD [2.5th percentile, 97.5th percentile].

[b] References: (Dennison et al. 2003); (Haddad et al. 1999); (Bois and Paxman 1992).

[c] References: (Tardif et al. 1997); (Haddad et al. 1999); (Dennison et al. 2003).

[d] References: (Tardif et al. 1997); (Haddad et al. 1999).

s

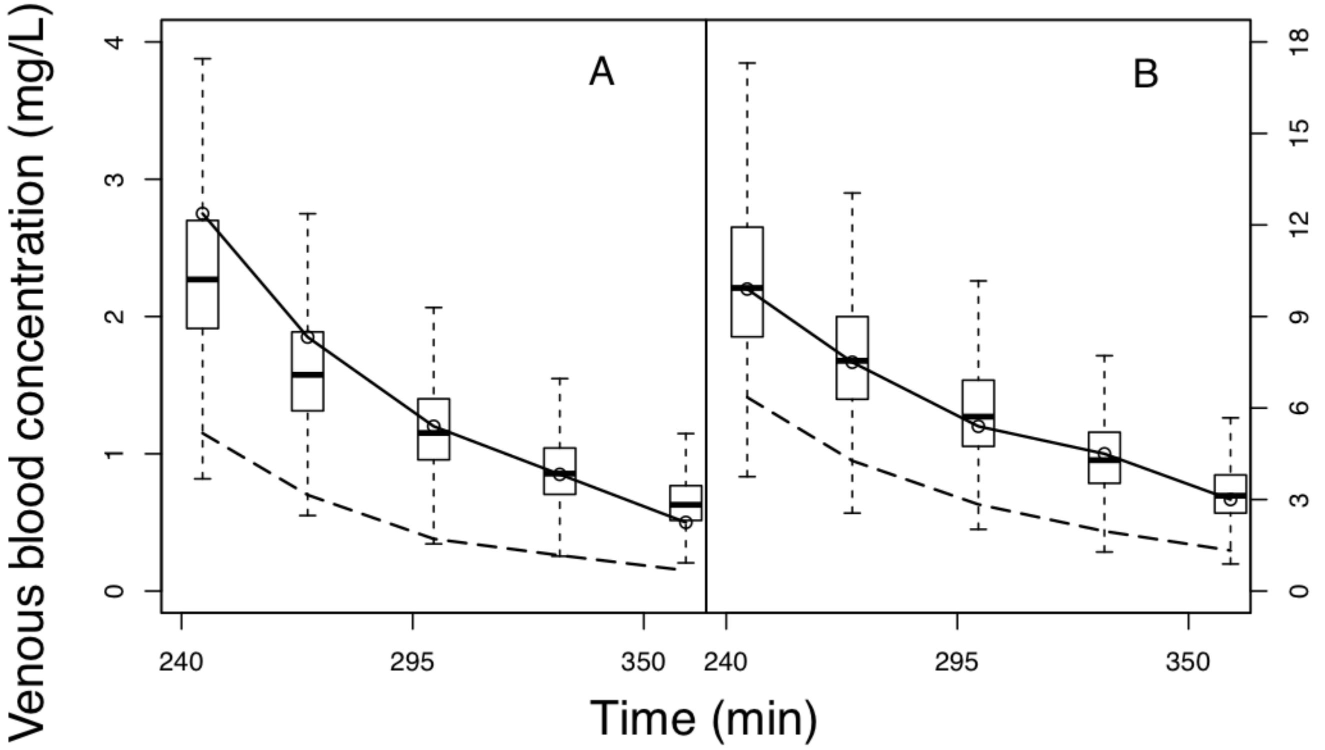

Figure S1: Blood kinetics of toluene (A) and *m*-xylene (B) after exposure to a binary mixture of 100 ppm of toluene and 200 ppm of *m*-xylene. The circles and solid lines show the mean experimental data for four rats (Tardif, 1996). The dashed lines show the blood kinetics of either toluene or *m*-xylene after single substance exposure to 100 ppm or 200 ppm in the air, respectively. The box plots display the inter-quartile range of the type I global model predictions (without any fitting to the mixture data).

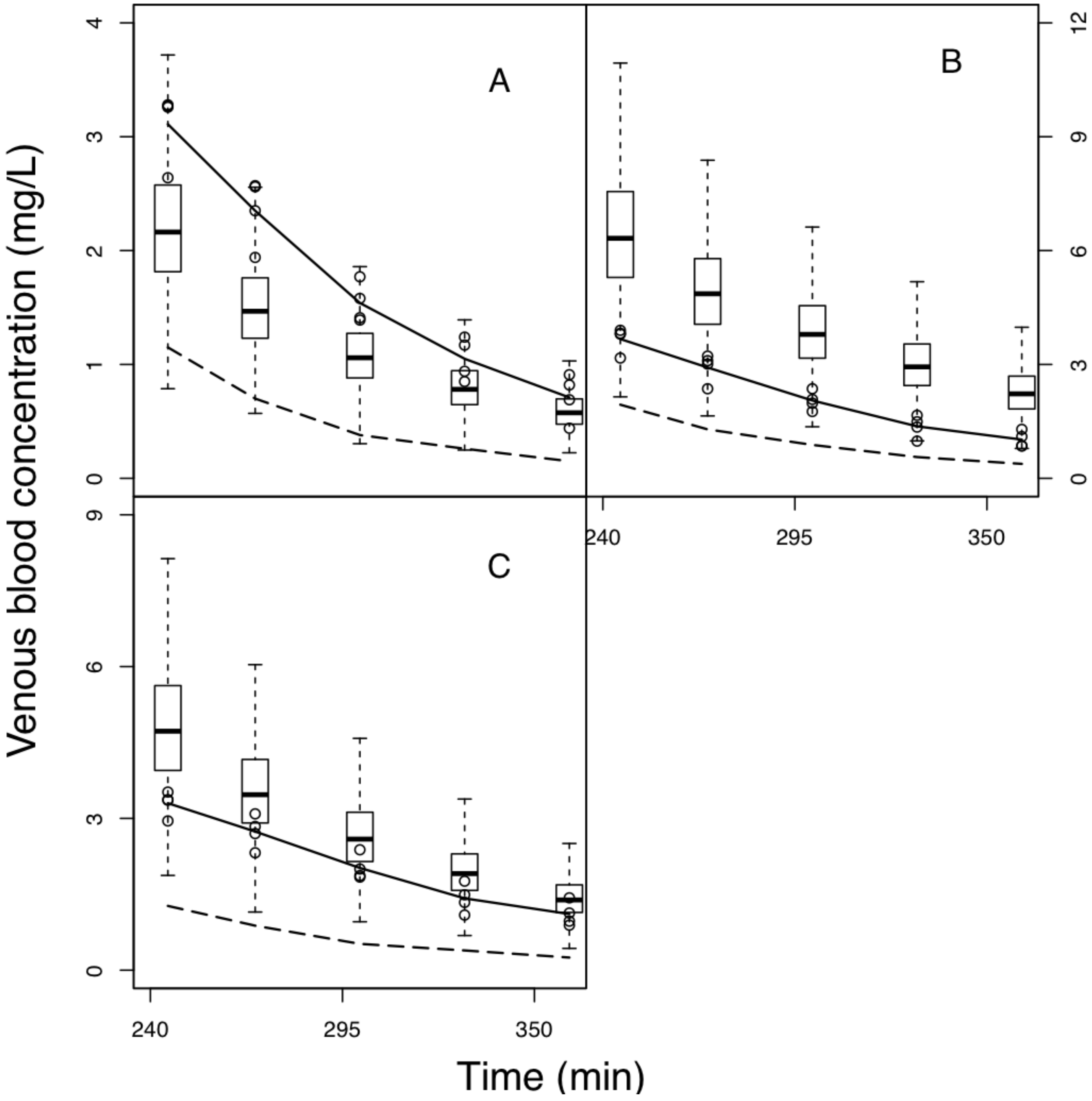

Figure S2: Blood kinetics of toluene (A), ethylbenzene (B) and *m*-xylene (C) after exposure to a ternary mixture of 100 ppm of each. The circles show the experimental data for four rats (Tardif, 1996) and the solid line gives their mean. The dashed lines show the blood kinetics of either T, E or X after single exposure to 100 ppm of the substance in the air. The box plots display the inter-quartile range of the type I global model predictions (without any fitting to the mixture data).

References

Bois FY, Paxman D. 1992. An analysis of exposure rate effects for benzene using a physiologically based pharmacokinetic model. Regulatory Toxicology and Pharmacology 15:122-136.

Dennison JE, Andersen ME, Yang RS. 2003. Characterization of the pharmacokinetics of gasoline using PBPK modeling with a complex mixtures chemical lumping approach. Inhalation Toxicology 15:961-986.

Haddad S, Tardif R, Charest-Tardif G, Krishnan K. 1999. Physiological modeling of the toxicokinetic interactions in a quaternary mixture of aromatic hydrocarbons. Toxicology and Applied Pharmacology 161:249-257.

Tardif R, Charest-Tardif G, Brodeur J. 1996. Comparison of the influence of binary mixtures versus a ternary mixture of inhaled aromatic hydrocarbons on their blood kinetics in the rat. Archives of Toxicology 70:405-413.

Tardif R, Charest-Tardif G, Brodeur J, Krishnan K. 1997. Physiologically Based Pharmacokinetic Modeling of a Ternary Mixture of Alkyl Benzenes in Rats and Humans. Toxicology and Applied Pharmacology 144:120-134.
